# Safety and immunogenicity of SARS-CoV-2 vaccine MVC-COV1901 in Taiwanese adolescents: a randomized phase 2 trial

**DOI:** 10.1038/s41541-022-00589-4

**Published:** 2022-12-16

**Authors:** Luke Tzu-Chi Liu, Cheng-Hsun Chiu, Nan-Chang Chiu, Boon-Fatt Tan, Chien-Yu Lin, Hao-Yuan Cheng, Meei-Yun Lin, Chia-En Lien, Charles Chen, Li-Min Huang

**Affiliations:** 1 Medigen Vaccine Biologics Corporation, Taipei, Taiwan; 2 Department of Pediatrics, Chang Gung Children’s Hospital, Chang Gung University College of Medicine, Taoyuan City, Taiwan; 3 Department of Pediatrics, MacKay Children’s Hospital, Taipei City, Taiwan; 4 Department of Pediatrics, National Taiwan University Hospital Hsinchu Branch, Hsinchu County, Taiwan; 5 Department of Pediatrics, Hsinchu MacKay Memorial Hospital, Hsinchu City, Taiwan; 6 Institute of Public Health, National Yang-Ming Chiao Tung University, Taipei City, Taiwan; 7 Department of Pediatrics, National Taiwan University Hospital and College of Medicine, National Taiwan University, Taipei, Taiwan

**Keywords:** Viral infection, Recombinant vaccine, Protein vaccines

## Abstract

Adolescents and children play an important role in SARS-CoV-2 transmission and epidemiology. MVC-COV1901 is a subunit SARS-CoV-2 vaccine based on stabilized spike protein adjuvanted with CpG 1018 and aluminum hydroxide that has received emergency use approval (EUA) for adults in Taiwan. In this study, we have investigated the safety and immunogenicity of two doses of MVC-COV1901 in adolescents. Healthy adolescents from the age of 12–17 years were randomly assigned to receive two intramuscular doses of either MVC-COV1901 or placebo at 28 days apart. Adverse events were mostly mild and were similar in MVC-COV1901 and placebo groups, with the most commonly reported adverse events being pain/tenderness and malaise/fatigue. All immunogenicity endpoints in the adolescent group were non-inferior to the endpoints seen in the young adult and placebo groups. The results here advocate the use of MVC-COV1901 in adolescents in the ongoing efforts to control the pandemic.

**ClinicalTrials.gov registration**: NCT04951388.

## Introduction

SARS-CoV-2 is the causative viral agent of COVID-19, an ongoing worldwide outbreak of pneumonia-like respiratory disease^1^. As of October 2022, over 625 million confirmed cases and 6.5 million deaths due to COVID-19 have been reported globally^2^. It is now clear that COVID-19 may become an endemic disease, as the rapid rate of natural viral evolution and emergence of novel variants do not permit the extinction of existing viral variants by antiviral treatments and vaccine-induced immunity before new variants arise^3,4^. Consequently, existing vaccines and treatments may have to be regularly redesigned to ensure their ability to optimally interact with and suppress new variants with mutations that reduce efficacy. This issue has recently been highlighted by the Omicron variant, which is more infectious than the prior Delta variant and therefore more difficult to eradicate^5^. In addition, this variant contains a new constellation of mutations that reduces the efficacy of at least some current treatments^6,7^.

There are also social and regulatory factors that complicate the eradication of COVID-19, such as vaccine hesitancy/resistance and stringent safety regulations for pediatric immunization that can delay clinical trial timelines^8^. This issue is now of more importance than ever because the omicron variant appears to be particularly infectious in younger populations^9^. While adolescents generally experience milder symptoms, the lingering sequelae of long COVID can be devastating to their health and development^10^. To date, the recently-approved Pfizer-BioNTech and Moderna vaccines are the only widely used vaccines for adolescents and children^11^. As a result, there remains a lag between vaccination rates in children/adolescents compared to adults. This can be a critical gap in COVID-19 transmission control. For example, as of October 2022 in the US, only 60.8% of adolescents aged 12–17 are fully vaccinated compared to 77.9% of adults over the age of 18^12^. Similarly, in Europe, 83.4% of adults are fully vaccinated compared to 71.3% of adolescents aged 15–17 and 37.5% of 10–14 years old as of October 12, 2022^13^. Therefore, the need to address adolescent vaccination is especially crucial in low- and lower-middle-income countries to address vaccine inequality and disrupt virus transmission.

MVC-COV1901 is a protein-based subunit vaccine comprising S-2P protein, a prefusion stable form of the spike protein of SARS-CoV-2 and toll-like receptor 9 agonist CpG 1018 and aluminum hydroxide as adjuvants^14^. MVC-COV1901 has been approved for emergency use authorization (EUA) in Taiwan for the prevention of COVID-19 in adults above the age of 18^11,15^. However, the safety and immunogenicity of MVC-COV1901 have yet to be explored in the pediatric population. To address this unmet need, the phase 2 trial described here explored the safety profile of two doses of MVC-COV1901 in adolescents 12–17 years of age and the immunogenicity of MVC-COV1901 in adolescents compared to young adults 20–30 years old.

## Results

### Study design and demographics

**Fig. 1:**
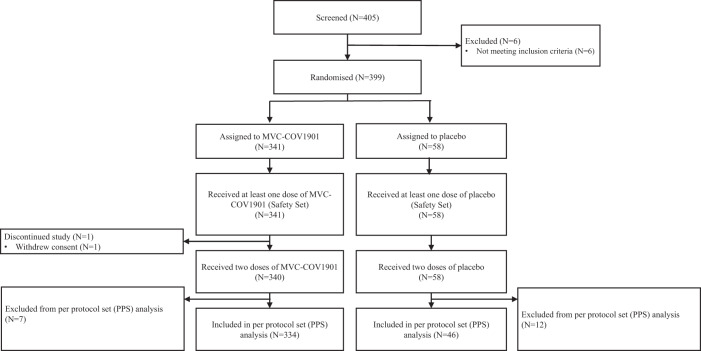
CONSORT Flow diagram of the study. Participants were enrolled from July 21, 2021 to December 22, 2021. The safety population included all participants who received at least one dose of the MVC-COV1901 vaccine or placebo. The per protocol set (PPS) population consisted of participants who received the two doses of MVC-COV1901 or placebo as scheduled, and were seronegative at baseline with anti-nucleocapsid (anti-N) antibodies negative at Visit 2 and Visit 6, and did not have a major protocol deviations. Detailed reasons for exclusion from the PPS can be found in Supplementary Table 2.

Between July 21, 2021, and December 22, 2021, a total of 405 adolescents have screened, 399 of which were deemed eligible and randomized at a 6:1 ratio to receive either MVC-COV1901 (*N* = 341) or placebo (*N* = 58) (Fig. 1, Supplementary Tables 1 and 2). To evaluate the immunogenicity of vaccination with MVC-COV1901 in adolescents of age 12–17 years, immunogenicity parameters were compared between this age group and young adults of age 20–30 years (*N* = 210). The demographics of the MVC-COV1901 and placebo groups and two age groups are summarized in Tables 1 and 2, respectively. Notably, there were a higher percentage of male in the MVC-COV1901 group and a higher percentage of female than male in the placebo group (*p* < 0.05, Chi-square test) (Table 1). There were no particular biases between the adolescent, and young adult groups except for the age and body mass index (BMI) group, which were significantly different (*p* < 0.0001, Mann–Whitney *U* test) between the two due to the age group differences as well as differences in interpretation of BMI in adults and children (Table 2).

**Table 1:** Summary of demographics of the participants who received study intervention (safety set).

	MVC-COV1901 (*N* = 341)	Placebo (*N* = 58)	All subjects (*N* = 399)	*P*-value
Age				0.6045 (Mann–Whitney *U* test)
Mean (SD)	14.4 (1.65)	14.2 (1.48)	14.3 (1.62)	
Min–Max	12.0–17.0	12.0–17.0	12.0–17.0	
Gender, *n* (%)				0.0429 (Chi-square test)
Male	190 (55.7)	24 (41.4)	214 (53.6)	
Female	151 (44.3)	34 (58.6)	185 (46.4)	
Race, *n* (%)				
Asian	341 (100.0)	58 (100.0)	399 (100.0)	
Non-Asian	0	0	0	
BMI (kg/m^2^),				0.3035 (Mann–Whitney *U* test)
Mean (SD)	21.2 (4.2)	21.9 (4.5)	21.3 (4.2)	
Min–Max	15.2–42.3	15.3–32.3	15.2–42.3	
BMI group, *n* (%)				0.0667 (Fisher’s exact test)
<30 kg/m^2^	330 (96.8)	53 (91.4)	383 (96.0)	
≥30 kg/m^2^	11 (3.2)	5 (8.6)	16 (4.0)	
HIV antibody, *n* (%)	0	0	0	
Pre-vaccination neutralizing antibody against wild type SARS-CoV-2 (≥8), *n* (%)	4 (1.2)	1 (1.7)	5 (1.3)	0.5460 (Fisher’s exact test)
Pre-vaccination anti-SARS-CoV-2 IgG (≥100), *n* (%)	10 (2.9)	2 (3.4)	12 (3.0)	0.6888 (Fisher’s exact test)
Comorbidity, *n* (%)				
Any	7 (2.1)	1 (1.7)	8 (2.0)	
Cardiovascular disease	0	0	0	
Cerebrovascular disease	0	0	0	
COPD	0	0	0	
Liver cirrhosis	0	0	0	
Malignancy	1 (0.3)	0	1 (0.3)	
HbAlc higher than 5.7% DCCT	6 (1.8)	1 (1.7)	7 (1.8)	

*N* number of participants in the population; *n* number of participants with available data, used as the denominator for percentage calculation; *SD* standard deviation.

**Table 2:** Summary of demographics of adolescent and young adults for immunogenicity comparison (PPS subset).

	Adolescent (12-17 years) (*N* = 334)	Young adults (20-30 years) (*N* = 210)	All subjects (*N* = 399)	*P*-value
Age				<0.0001 (Mann–Whitney *U* test)
Mean (SD)	14.4 (1.65)	25.9 (2.74)	18.8 (6.02)	
Min–Max	12.0–17.0	20.0–30.0	12.0–30.0	
Gender, *n* (%)				0.1521 (Chi-square test)
Male	188 (56.3)	105 (50.0)	293 (53.9)	
Female	146 (43.7)	105 (50.0)	251 (46.1)	
Race, *n* (%)				
Asian	334 (100.0)	210 (100.0)	544 (100.0)	
Non-Asian				
BMI (kg/m^2^),				<0.0001 (Mann–Whitney *U* test)
Mean (SD)	21.2 (4.19)	23.6 (4.09)	22.1 (4.31)	
Min–Max	15.2 ~ 42.3	14.4 ~ 40.5	14.4 ~ 42.3	
BMI group, *n* (%)				0.0076 (Chi-square test)
<30 kg/m^2^	323 (96.7)	192 (91.4)	515 (94.7)	
≥30 kg/m^2^	11 (3.3)	18 (8.6)	29 (5.3)	
HIV antibody, *n* (%)	0	1 (0.5)	1 (0.2)	0.3860 (Fisher’s exact test)
Pre-vaccination neutralizing antibody against wild-type SARS-CoV-2 (≥8), *n* (%)	0	0	0	
Pre-vaccination anti-SARS-CoV-2 IgG (≥100), *n* (%)	9 (2.7)	9 (4.3)	18 (3.3)	0.3125 (Chi-square test)
Comorbidity, *n* (%)				
Any	6 (1.8)	6 (2.9)	12 (2.2)	
Cardiovascular disease	0	1 (0.5)	1 (0.2)	
Cerebrovascular disease	0	0	0	
COPD	0	0	0	
Liver cirrhosis	0	0	0	
Malignancy	0	0	0	
HbAlc higher than 5.7% DCCT	6 (1.8)	5 (2.4)	11 (2.0)	

*N* number of participants in the population; *n* number of participants with available data, used as the denominator for percentage calculation; *SD* standard deviation.

### Safety of MVC-COV1901 in adolescents

**Fig. 2:**
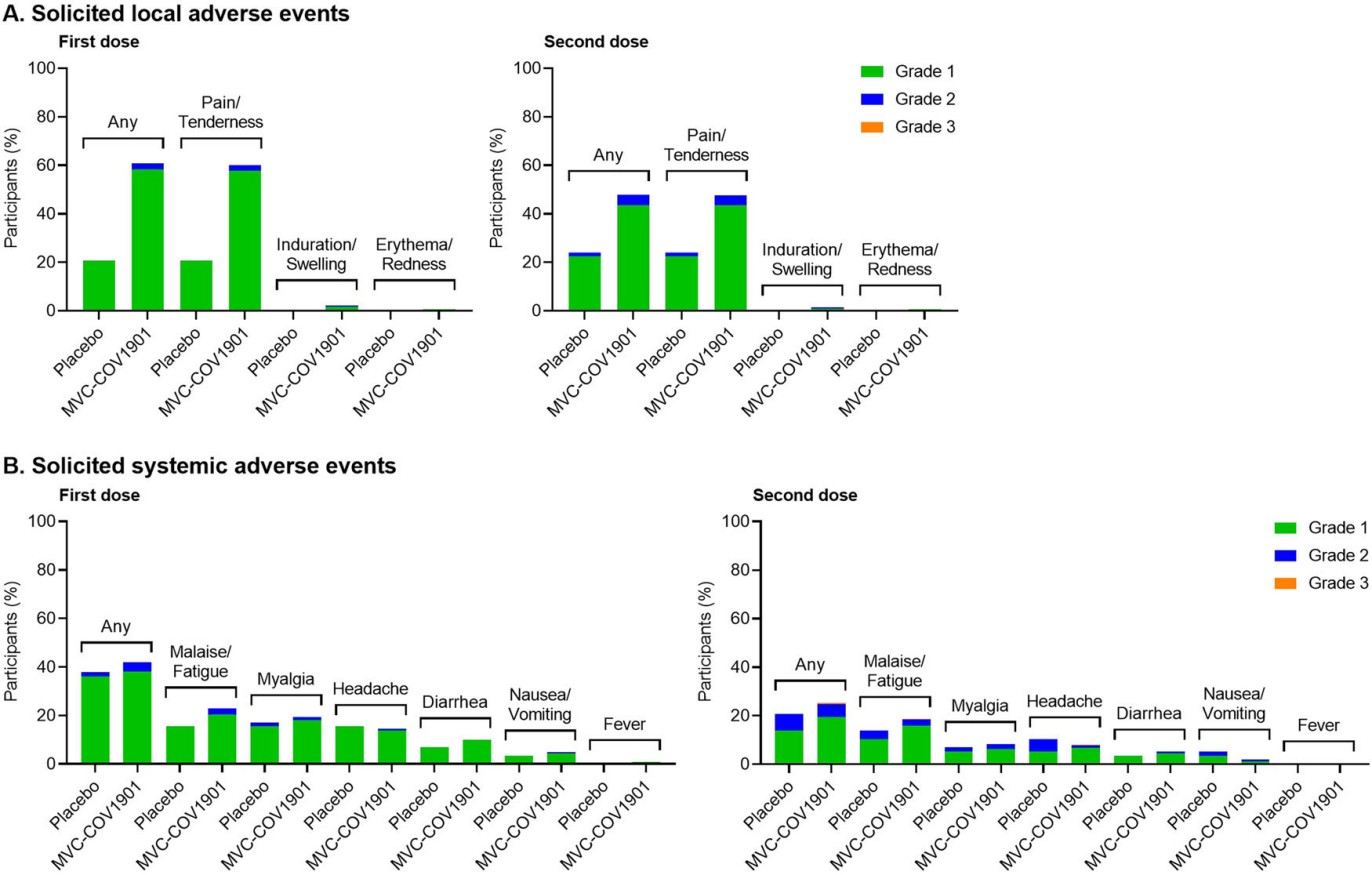
Adverse events in adolescents after administration of a first or second dose of MVC-COV1901. Solicited local (**A**) and systemic (**B**) adverse events were graded as mild (grade 1), moderate (grade 2), or severe (grade 3).

All individuals who received at least one of the two vaccinations were evaluated for safety analysis. The incidences of solicited adverse events (AEs) after the first and second doses of vaccination are summarized in Fig. 2 and Supplementary Table 3. The solicited AEs were mostly mild, and the most frequently reported first-dose local AE was pain/tenderness in the MVC-COV1901 treatment groups, where 69.8% of individuals were affected, compared to 32.8% in the placebo group. The most common systemic AE was malaise/fatigue, which occurred in 30.8% and 24.1% of participants in MVC-COV1901 and placebo groups, respectively. Only two subjects with grade 3 events were recorded: One for malaise/fatigue and one for headache (Supplementary Table 3).

Except for the absence of fever and a slightly increased incidence of headache, individuals in the placebo group also experienced all systemic AEs but at marginally reduced rates. A total of 26.1% of all participants experienced unsolicited AEs, where the numbers for the placebo group (34.5%) were slightly higher than that for the MVC-COV1901 group (24.6%) (Supplementary Table 3). Eight individuals presented unsolicited AEs equal to or greater than Grade 3, but only one event (urticaria) in the placebo group was deemed to be related to intervention by the investigator according to the assessment of causality in the protocol. No cases of serious AEs (SAEs), AEs of special interest (AESI), vaccine-associated enhanced disease (VAED), and deaths were reported.

### Immunogenicity of MVC-1901 in adolescents

**Fig. 3:**
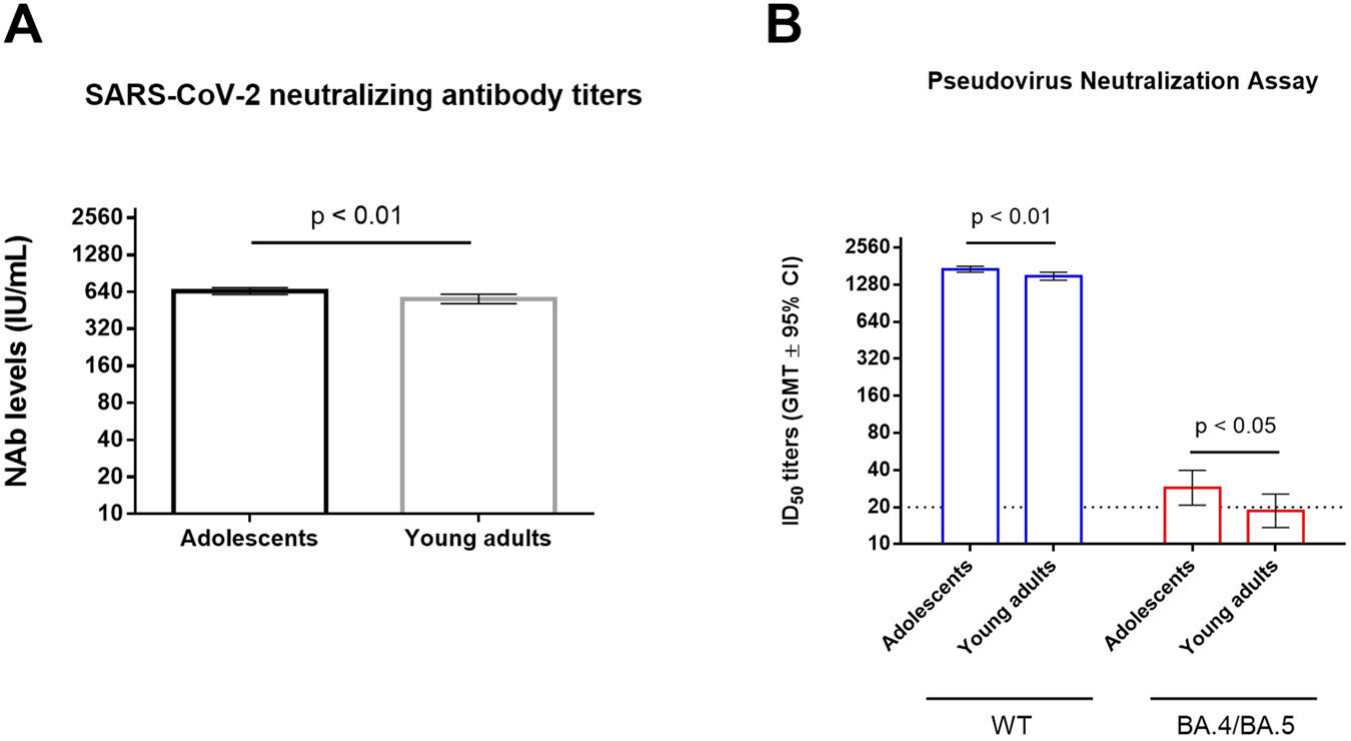
Neutralizing assay results of adolescent and young adult groups. Twenty-eight days after the second dose, serum samples were taken and subjected to the (**A**) live-SARS-CoV-2 and (**B**) pseudovirus neutralization assays. The ancestral Wuhan (WT) strain was used in both the live virus and pseudovirus assay, while Omicron BA.4/BA.5 was only used in the pseudovirus assay. The results are expressed as symbols representing GMT, and error bars represent 95% confidence intervals. The dotted line in (**B**) represents the limit of detection of pseudovirus neutralization assay (20). Statistical significance was calculated using the Mann–Whitney *U* test.

Primary immunogenicity endpoint assessment was performed on Day 57 by comparing geometric mean titer (GMT) and seroconversion rate (SCR, defined as at least a fourfold increase from the baseline titer) between the adolescent and young adult groups. After conversion to the WHO International Units (IU/mL), the GMTs of adolescent and young adults were 648.5 [95% CI: 608.6–690.9] and 559.5 [95% CI: 512.1–611.3], respectively (Fig. 3A, Table 3). The GMT ratio was 1.16 [95% CI: 1.04–1.29], which was higher than the value of 0.67, thus demonstrating the non-inferiority of immunogenicity of MVC-COV1901 in adolescents compared to young adults (Table 3). The SCR based on neutralization assay was 100% in both adolescent and young adult groups (Table 4). Therefore, both groups were similarly completely seroconverted by two doses of MVC-COV1901, again demonstrating the non-inferiority of MVC-COV1901 in the adolescent group. In addition, to explore the immunogenicity of two doses of MVC-COV1901 against the Omicron variant, pseudovirus neutralization assays with the original Wuhan strain (WT) and BA.4/BA,5 Omicron subvariant were performed. There were dramatic decreases in GMTs against the Omicron variant compared to the WT, which had lowered nearly the detection limit for the adolescent group and below the detection limit for the young adult group (Fig. 3B).

**Table 3:** Summary of non-inferiority of live SARS-CoV-2 neutralizing antibody titers between adolescent and young adults who received MVC-COV1901 (in IU/mL).

Per protocol set (PPS) Visit 6 (Day 57)	Adolescents of MVC-COV1901 (*N* = 334)	Young Adults of MVC-COV1901 (*N* = 210)	Ratio adolescent/young adults	*P*-value
Median (IQR)	643.62 (980.4)	444.87 (498.45)	–	–
Q1–Q3	429.67–964.11	397.47–916.71	–	–
Min–Max	136.20–3466.39	120.46–6976.30	–	–
GMT	648.47	559.54	1.16	*P* < 0.05^a^
95% CI	608.62–690.93	512.05–611.34	1.04–1.29	

^a^Two sample *t*-test.

**Table 4:** Summary of non-inferiority of live SARS-CoV-2 neutralizing antibody seroconversion rate (SCR) between adolescents (12–<18 years) and young adults (20–30 years) who received MVC-COV1901.

Per protocol set (PPS) Visit 6 (Day 57)	Adolescents of MVC-COV1901 (*N* = 334)	Young Adults of MVC-COV1901 (*N* = 210)	Difference adolescent/young adults	*P*-value
SCR, *n* (%)	334 (100%)	210 (100%)	−0.0%	N/A
95% CI^a^	98.90–100.00%	98.26–100.00%	−0.00% to 0.00%	

^a^CI calculated using a normal approximation for the binomial proportion of #.

For secondary immunogenicity endpoint assessment, only interim results up to Visit 6 are available. The levels of neutralizing antibody and anti-spike immunoglobulin G (IgG) were similar at baseline level at Visit 2 for MVC-COV1901 and placebo groups (Supplementary Table 5). After two doses of vaccination, the GMT in the MVC-COV1901 group increased to 648.5 [95% CI: 608.6–690.9], while the GMT for placebo remained at a baseline level, thus yielding a ratio of 115.2 [95% CI: 95.4–139.2] between MVC-COV1901 and placebo GMT levels (Supplementary Table 5). Similar results were seen from the IgG assay, in which the MVC-COV1901 group already had a GMT of 136.0 [95% CI: 122.9–150.6] at Visit 4 that further increased to 1632.0 [95% CI: 1514.9–1758.0] at Visit 6. The GMT ratios of the MVC-COV1901 group to the placebo group were 27.8 [95% CI: 24.3–31.7] and 254.4 [95% CI: 167.1–387.3] at Visits 4 and 6, respectively (Supplementary Table 6). In terms of SCR, the MVC-COV1901 group was completely seroconverted by Visit 6 as assessed by neutralization assay or IgG (Supplementary Table 7). Notably, after only one dose of MVC-COV1901, 97.3% [95% CI: 95.0–98.8] of MVC-COV1901 recipients were already seroconverted by the time they were to receive the second dose (Visit 4) (Supplementary Table 7).

## Discussion

Here, we have investigated two doses of the SARS-COV-2 subunit vaccine MVC-COV1901 for safety and immunogenicity in adolescents between 12 and 17 years of age. The study design was largely guided by the results from a prior large phase 2 trial of the vaccine in adults. Thus, we believe that two intramuscular doses of MVC-COV1901 at 28 days apart and immunogenicity analysis 28 days after the second shot on Day 57 were optimal for properly assessing the vaccine in the younger age group studied here.

Safety data of MVC-COV1901 were similar overall to placebo for systemic AEs (Fig. 2, Supplementary Table 3). Only local solicited AEs had greater incidences in the vaccine treatment group compared to the placebo, but local solicited AEs were grade 1 except for 5.5% of participants who experienced grade 2 symptoms after any dose of either MVC-COV1901 or placebo (Fig. 2, Supplementary Table 3). The AEs are similar to those observed for adult participants in phase 2 clinical trial and also similar to the V-Watch vaccine safety monitoring program data collected by the Taiwan Centers for Disease Control, in which pain and fatigue/malaise were the most commonly reported AEs and very few incidences of fever^15,16^. Overall, therefore, the safety data suggest a highly favorable safety profile for the adolescent age group comparable to the adults.

A consistent picture emerged where immunogenicity was as robust in the adolescent age group as in the larger phase 2 trial. Essentially, the primary study endpoints of live virus neutralizing titers and SCRs were non-inferior when compared with young adults (Tables 3 and 4). Similarly, the secondary endpoints of live virus-neutralizing antibody titers and anti-spike IgG titers increased at least two orders of magnitude compared to the placebo at 28 days after the second dose (Supplementary Table 6). As antibody titers diminish over time, the availability of full results up to Day 209 will be useful in gauging the degree of waning immunity in adolescents, as compared to our phase 1 extension results with three doses of MVC-COV1901 in adults, which tracked antibody decay up to Day 209 prior to administration of the third dose as booster^17^. We have previously performed a post hoc analysis of a phase 2 clinical trial and found a negative association between increasing antibody titers and decreasing age^18^. Thus, unsurprisingly, the adolescent group developed greater neutralizing antibodies and IgG titers than the young adult group (Table 3). The observation that two doses of MVC-COV1901 had minimal effect on neutralization of BA.4/BA.5 Omicron subvariant is unsurprising as other studies showed that two doses of other approved vaccines, such as mRNA-1273 and BNT162b2 were ineffective against various Omicron subvariants^6,7,19^. We and others have shown that homologous or heterologous booster doses improve immunogenicity against the Omicron subvariants^7,17,19^. Thus, the lowered immunogenicity against the Omicron subvariants after only two doses of vaccine is not a cause for alarm as most of the population in the high-income countries have already been boosted. However, this also laid bare the fact of vaccine inequality that could contribute to the further spread and appearance of new variants in the low- and-middle-income countries where vaccination is scarce.

Several studies of mRNA COVID-19 vaccines in adolescents and children were completed and contributed to their approval for use in the younger populations in the US and Europe. Moderna mRNA-1273 was non-inferior in terms of GMT ratio and seroconversion in adolescents aged 12–17 years versus young adults aged 18–25 years and had an efficacy of 93.3% according to the US CDC definition of COVID-19 with an onset of 14 days after the second dose^20^. In another study with mRNA-1273, two doses of mRNA-1273 invoked neutralization responses against the Omicron variant pseudovirus that resulted in numerically superior GMT values in adolescents and children compared to adults^21^. BNT162b2 also demonstrated a higher neutralizing antibody response in the 12- to 15-year-old adolescents than in adults and had an observed efficacy of 100% according to the definition of COVID-19 cases with an onset of 7 or more days after the second dose^22^. In all, these external results and our results not only clearly showed that COVID-19 vaccines are suitable for adolescents and children, but that they also generate better humoral immunity than in adults. Thus, the use of vaccines in the young population should help to stop this critical chain of transmission. Taken together, the results of this and other studies have shown that in adolescents and children, vaccination can grant superior antibody immune responses to SARS-CoV-2 than that of adults.

Limitations apply to this study. The main limitation is that this is a phase 2 study where efficacy was not assessed directly. Therefore, immunogenicity was the result of interest. Although earlier studies suggested the association between immunogenicity and protection against infection, correlates of protection for SARS-CoV-2 were not established, particularly in the era of Omicron^23,24^. Due to ethnic homogeneity and limited ethnic diversity in Taiwan, our study was only able to enroll local Taiwanese people (Han Chinese and possibly some Aboriginal mixture), and this should be taken into consideration when the vaccine is used in regions with other or more diverse ethnic makeup. The BMI between adolescents and young adults was significantly different; however, BMI could not be directly compared because interpretation is different for children and adults, and interpretation of BMI in children is age and sex-specific^25^. The sample size in this study may not be sufficiently large enough to detect rare but SAEs such as thrombosis and myocarditis. However, according to Taiwan Centers for Disease Control, incidences of SAEs are extremely rare compared to other authorized COVID-19 vaccines^26^.

The data presented here make a case for further clinical assessment. Secondly, even though most safety events were grade 1, a small number of grade 3 events were observed that need to be evaluated in a greater study population. Notably, though, no grade 4 events were observed, and there were also no cases of AESI and VAED. Other limitations relate to questions regarding the evolution of COVID-19 over the last year that clinical trials are in the process of catching up with. These questions include the duration of the neutralizing antibody response over several months after the second dose, the suitability of any given vaccine for current and future virus variants, and if there is any additional protection afforded by booster shots. As Taiwan has started administering booster doses for adults, including MVC-COV1901, future studies will investigate the suitability of MVC-COV1901 as a homologous or heterologous booster after primary vaccination of adolescents.

## Methods

### Study design and participants

This is an interim analysis of an ongoing phase 2, prospective, double-blind (investigator/site staff and participants), and multi-center study to assess the safety and immunogenicity of MVC-COV1901 in adolescents aged 12–17 years. The study sites included five hospitals/medical centers in Taiwan. Eligible participants were those aged 12 (inclusive) to 17 at the time of randomization, with BMI at or above the third percentile according to World Health Organization (WHO) at the screening visit, and lack of travel within 14 days of screening and lack of any oversea traveling throughout the study period. Finally, the participant and/or the participant’s legal representative must have provided written informed consent. Additional criteria applied to females only: negative pregnancy test, non-childbearing potential, or, if with childbearing potential, abstinence or agreement to use medically effective contraception from 14 days before screening to 30 days following the last injection of the study intervention. A full list of inclusion and exclusion criteria can be found in the study protocol in the Appendix.

The trial protocol and informed consent form were approved by Taiwan Food and Drug Administration (TFDA) and the ethics committees at the conducting sites: Mackay Memorial Hospital Hsinchu (Hsinchu City), Chang-Gung Memorial Hospital Linkou (New Taipei City), Mackay Memorial Hospital (Taipei City), National Taiwan University Hospital Hsinchu (Hsinchu City), and National Taiwan University Hospital (Taipei City). An Independent Data Monitoring Committee (IDMC) was established to monitor data safety and trial conduct. The study complies with the protocol and statistical analysis plan. This trial was conducted in accordance with the principles of the Declaration of Helsinki and Good Clinical Practice (GCP) guidelines.

### Randomization and blinding

All eligible participants were randomized to receive either MVC-COV1901 or a placebo in a 6:1 ratio. All participants were centrally assigned to randomized study interventions using an interactive web response system (IWRS). Before the study was initiated, the log-in information and directions for the IWRS were provided to each site.

This was a double-blind study in which participants and investigators were blinded to the study intervention. The IWRS was programmed with blind-breaking instructions, and in case of an emergency, the investigator had the sole responsibility for determining if unblinding of participants’ intervention assignment was warranted. For the interim analysis, an independent, unblinded team of the CRO consisting of personnel representing relevant functions, including statistics, programming, and report writing, was involved in the activity of the interim analysis. All activities of the unblinded team were separated from the main blinded team of the study. The Sponsor was blinded until the time of the interim analysis.

As MVC-COV1901 and placebo were visually distinct in their color of appearance, the investigator had assigned unblinded qualified personnel who were not involved in any other aspect of the study to handle the preparation, dispensing, administration, and accountability of the study intervention. Study-specific training was provided at the study site to ensure treatment blinding of all other study staff and the participants.

### Procedures

The study schedule is outlined in Supplementary Fig. 1. The participants were intramuscularly administered in the deltoid region with two doses of either MVC-COV1901 or placebo (saline) on Day 1 (Visit 2) and Day 29 (Visit 4). The vaccine MVC-COV1901 and placebo were produced at Medigen Vaccine Biologics Zhubei facility (Hsinchu County, Taiwan) in compliance with current good manufacturing practices. MVC-COV1901 contained 15 μg S-2P (stabilized prefusion form of SARS-CoV-2 spike protein), 750 μg CpG 1018, and 375 μg aluminum hydroxide at a final volume of 0.5 mL per dose. The placebo contained 0.5 mL of saline solution per dose. Participant samples were collected during six on-site visits: on Days—28 to 0 (Screening Visit), 1 (first vaccination), 29 (second vaccination), 57, 119, and 209. Safety data was monitored by three telephone calls on Days 8, 36, and 85.

For safety analysis, vital signs were assessed before and after each injection. Participants were observed for at least 30 min after each injection to identify any immediate AEs. All participants who received at least one of the two doses of MVC-COV19 scheduled for Day 1 and Day 29 were evaluated for safety up to Day 119 (Visit 8) by assessing the following endpoints: solicited local AEs (up to 7 days after each dose of study intervention), solicited systemic AEs (up to 7 days after each dose of study intervention), unsolicited AEs (up to 28 days after each dose of study intervention), AESI, VAED and SAEs. Solicited AEs were defined as AEs which occurred within 7 days after each dose of study intervention, including local events: pain/tenderness, erythema/redness, and induration/swelling; systemic events: fever, malaise/fatigue, myalgia, headache, nausea/vomiting, and diarrhea. Unsolicited AEs are defined as any untoward medical events other than solicited AEs which occurred within 28 days after each dose of the study intervention. The intensities of solicited and unsolicited AEs were graded using grading scales modified from the US FDA Guidance for Industry^27^.

To evaluate immunogenicity, live SARS-CoV-2 neutralization assay and anti-SARS-CoV-2 spike immunoglobulin (IgG) enzyme-linked immunosorbent assay (ELISA) were performed^15,17^. Briefly, serially twofold diluted sera were mixed with an equal volume of SARS-CoV-2 virus (hCoV-19/Taiwan/4/2020, GISAID accession ID: EPI_ISL_411927). The serum-virus mixture was incubated and then added to the plates containing Vero E6 cells, followed by further incubation. The neutralizing antibody titer was defined as the reciprocal of the highest dilution capable of inhibiting 50% of the cytopathic effect (50% inhibiting dilution, ID_50_), which was calculated using the Reed-Muench method. For anti-SARS-CoV-2 spike IgG ELISA, antigen-specific immunoglobulin titers to S-2P protein were evaluated in serum samples collected from participants. The detection and characterization of antigen-specific immunoglobulin were performed by a central laboratory using a validated ELISA method with plates coated with S-2P proteins^28^. Pseudovirus constructed from lentivirus with spike proteins of original Wuhan strain or Omicron (BA.4/BA.5) variant was used in pseudovirus neutralization assay^14^. Heat-inactivated serially diluted sera were mixed with an equal volume of pseudovirus (1000 transduction units) and incubated before adding to the plates with HEK293-hAce2 cells (1 × 10^4^ cells/well). The mixtures were incubated at 37 °C for 1 h before adding to the HEK293-hAce2 cells. One day after incubation with the cell culture, the culture medium was replaced with 100 μL of fresh medium. Cells were lysed at 72 h after transduction, and relative luciferase units were measured with Tecan i-control (Infinite 500).

For standardization of results, the GMTs of ID_50_ from the neutralization assay were converted to International Units (IUs/mL) in the following transformations defined experimentally: *y* = 1.5001 ∙ *x*^0.8745^ for the adolescent data and *y* = 0.4964 ∙ *x*^1.0334^ for the young adult data, where y is the value of IU/mL and *x* is the value of the GMT. Similarly, the GMTs from the ELISA assay were converted to Binding Antibody Units (BAUs/mL) as previously established by multiplying by a factor of 0.0912^15^.

### Outcomes

The primary safety outcome included the occurrence rate of solicited (local and systemic) AEs, unsolicited AEs, AESI, VAED, and SAE from Visit 2 (Day 1) to Visit 6 (28 days after the second dose of study intervention). The primary immunogenicity outcomes were neutralizing antibody titers and SCR against the live SARS-CoV-2 virus of MVC-COV1901 in adolescents as compared to young adult vaccinees at Visit 6 (Day 57).

The secondary safety outcome included the occurrence rate of ≥Grade 3 AE, AESI, VAED, and SAE over the whole study period, i.e., from Day 1 to 180 days after the second vaccination (Day 209). For secondary immunogenicity outcomes, comparisons of MVC-COV1901 against placebo were performed by measuring and expressing antigen-specific immunoglobulin titers and neutralizing antibody titers in samples taken at Visit 4 (28 days after the first dose of study intervention), Visit 6 (28 days after the second dose of study intervention), Visit 8 (90 days after the second dose of study intervention) and Visit 9 (209 days after the second dose of study intervention).

### Statistical analysis

Three hundred and ninety-nine participants were randomly assigned to study intervention. The sample size was powered to demonstrate that the immunogenicity of MVC-COV1901 in adolescents is non-inferior to that in young adults assessed by neutralizing antibody GMT and SCR 28 days after the second dose of the study intervention. Demographical and immunogenicity data of 210 young adults (20–30 years old, who were seronegative for SARS-CoV-2 neutralizing antibody at baseline and received MVC-COV1901), were randomly selected from a previous phase 2 trial for comparison^15^.

Safety was analyzed with the safety set population, which consisted of all randomized participants who received at least one dose of the study intervention. Immunogenicity was analyzed with the per-protocol set population, which consisted of the individuals who received the planned doses of randomized study intervention per schedule, were seronegative at baseline (neutralizing antibody titer < lower limit of detection (LoD) at Visit 2), anti-nucleocapsid (anti-N) antibodies negative at Visit 2 and Visit 6, and did not have a major protocol deviation that was judged to impact the critical or key study data.

Seroconversion was defined as at least a fourfold increase of post-study intervention antibody titers from the baseline titer or from half of the lower LoD if undetectable at baseline. Immunogenicity results were presented as GMTs, GMT ratios, and SCRs with two-sided 95% CIs.

The GMT ratio and two-sided 95% CIs were calculated by exponentiating the mean difference of the logarithms of the titers (the adolescent group cohort minus the young adult group) and the corresponding confidence intervals (based on the two samples *t*-test). For SCR, the chi-square test was used to compare the two treatment arms.

Immunogenicity primary endpoints were the non-inferiority of neutralizing antibody GMT ratio and SCR at 28 days after the second dose of MVC-COV1901 in adolescents compared to young adults. The adolescent group was claimed as non-inferior to the young adult group in the GMT treatment ratio when the lower bound of the two-sided 95% CI of the GMT treatment ratio is greater or equal to 0.67. The adolescent group was deemed as non-inferior to the young adult group in SCR treatment difference when the lower bound of the two-sided 95% CI of SCR treatment difference is greater or equal to −10%.

The secondary immunogenicity endpoints of the study intervention included the GMT, SCR, and GMT ratio of antigen-specific immunoglobulin titers and neutralizing antibody titers at Visit 4 (28 days after the first dose of study intervention), Visit 6 (28 days after the second dose of study intervention), Visit 8 (90 days after the second dose of study intervention) and Visit 9 (180 days after the second dose of study intervention). Only the results at Visit 4 and Visit 6 were available for the interim results. GMT ratio in the secondary immunogenicity endpoint was defined as the geometric mean of fold increase of post-study intervention titers over the baseline titers. Prism 6.01 (GraphPad) and its statistical package was used for statistical analysis.

### Reporting summary

Further information on research design is available in the Nature Research Reporting Summary linked to this article.

## Supplementary information

Supplemental data

REPORTING SUMMARY

## Data Availability

Data are available from the corresponding authors (C.C. and L.-M.H.) upon request.
