# Supplementary material for: Safety and immunogenicity of SARS-CoV-2 vaccine MVC-COV1901 in Taiwanese adolescents: a randomized phase 2 trial
Source: NPJ Vaccines. 2022 Dec 16;7:165. doi: 10.1038/s41541-022-00589-4 (PMC9755761; doi:10.1038/s41541-022-00589-4)
Supplement: Supplementary file 1 — Supplemental data [file 41541_2022_589_MOESM1_ESM.pdf]

Supplementary Figure 1. Timeline of the study

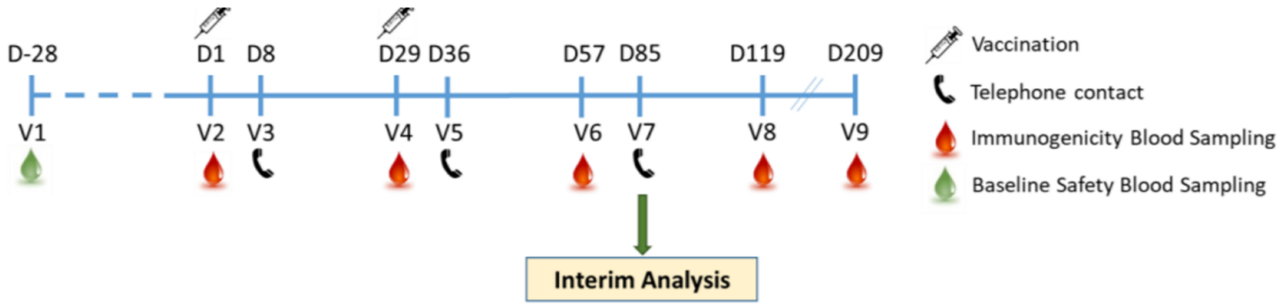

Supplementary Table 1. Summary of Screen failure

Number of Participants	Reason for screen failure
**1**	Exclusion 12:<br>Bleeding disorder considered a contraindication to intramuscular injection or phlebotomy.
**1**	Inclusion 2:<br>Body mass index (BMI) at or above the third percentile according to World Health Organization (WHO) BMI-for-age (Appendix 8: Section 10.8) at the Screening Visit.
**5**	Inclusion 4:<br>Participant is willing and able to comply with all required study visits and follow-up required by this protocol.

NB: One participant had two of the above reasons for screen failure, resulting in screen failure for a total of six participants.

Supplementary Table 2. Summary of Analysis Populations

	MVC-COV1901	Placebo	Total
**Safety Set**			
N	341	58	399
- PPS	334 (97.9%)	46 (79.3%)	380 (95.2%)
- Non-PPS	7 (2.1%)	12 (20.7%)	19 (4.8%)
- Received prohibited treatment before Visit 6	1 (0.3%)	7 (12.1%)	8 (2.0%)
- The immunogenicity sampling was not done (SARS-COV-2 antibody included) at Visit 6	1 (0.3%)	4 (6.9%)	5 (1.3%)
- The neutralizing antibody titer is not lower than lower limit of detection at Visit 2	4(1.2%)	1 (1.7%)	5 (1.3%)
- The result of anti-N is not negative at Visit 2	1 (0.3%)	0	1 (0.3%)
- The result of anti-N is not negative at Visit 6	1 (0.3%)	0	1 (0.3%)
- The subject only received one dose	1 (0.3%)	0	1 (0.3%)
- Violation of exclusion criterion 9 at Visit 1	1 (0.3%)	0	1 (0.3%)
- Violation of exclusion criterion 10 at Visit 1	1 (0.3%)	0	1 (0.3%)

NB: Participants may have two or more of the above reasons for exclusion.

Supplementary Table 3. Summary of solicited adverse events after each dosing

	After any dose, n (%)			After first dose, n (%)			After second dose, n (%)		
	MVC-COV1901 (N = 341)	Placebo (N = 58)	Total (N = 399)	MVC-COV1901 (N = 341)	Placebo (N = 58)	Total (N = 399)	MVC-COV1901 (N = 340)	Placebo (N = 58)	Total (N = 398)
**Any AEs**	265 (77.7)	31 (53.4)	296 (74.2)	240 (70.4)	26 (44.8)	266 (66.7)	182 (53.5)	16 (27.6)	198 (49.7)
Grade 1	225 (66.0)	26 (44.8)	251 (62.9)	221 (64.8)	25 (43.1)	246 (61.7)	155 (45.6)	12 (20.7)	167 (42.0)
Grade 2	38 (11.1)	5 (8.6)	43 (10.8)	19 (5.6)	1 (1.7)	20 (5.0)	25 (7.4)	4 (6.9)	29 (7.3)
Grade 3	2 (0.6)	0	2 (0.5)	0	0	0	2 (0.6)	0	2 (0.5)
**Any Solicited Local AEs**	238 (69.8)	19 (32.8)	257 (64.4)	207 (60.7)	12 (20.7)	219 (54.9)	163 (47.9)	14 (24.1)	177 (44.5)
Grade 1	217 (63.6)	18 (31.0)	235 (58.9)	199 (58.4)	12 (20.7)	211 (52.9)	148 (43.5)	13 (22.4)	161 (40.5)
Grade 2	21 (6.2)	1 (1.7)	22 (5.5)	8 (2.3)	0	8 (2.0)	15 (4.4)	1 (1.7)	16 (4.0)
**Pain/Tenderness**	238 (69.8)	19 (32.8)	257 (64.4)	205 (60.1)	12 (20.7)	217 (54.4)	162 (47.6)	14 (24.1)	176 (44.2)
Grade 1	218 (63.9)	18 (31.0)	236 (59.1)	197 (57.8)	12 (20.7)	209 (52.4)	148 (43.5)	13 (22.4)	161 (40.5)
Grade 2	20 (5.9)	1 (1.7)	21 (5.3)	8 (2.3)	0	8 (2.0)	14 (4.1)	1 (1.7)	15 (3.8)
**Induration/Swelling**	11 (3.2)	0	11 (2.8)	7 (2.1)	0	7 (1.8)	5 (1.5)	0	5 (1.3)
Grade 1	8 (2.3)	0	8 (2.0)	6 (1.8)	0	6 (1.5)	3 (0.9)	0	3 (0.8)
Grade 2	3 (0.9)	0	3 (0.8)	1 (0.3)	0	1 (0.3)	2 (0.6)	0	2 (0.5)
**Erythema/Redness**	4 (1.2)	0	4 (1.0)	2 (0.6)	0	2 (0.5)	2 (0.6)	0	2 (0.5)
Grade 1	4 (1.2)	0	4 (1.0)	2 (0.6)	0	2 (0.5)	2 (0.6)	0	2 (0.5)
**Any Solicited Systemic AEs**	162 (47.5)	26 (44.8)	188 (47.1)	143 (41.9)	22 (37.9)	165 (41.4)	86 (25.3)	12 (20.7)	98 (24.6)

	After any dose, n (%)			After first dose, n (%)			After second dose, n (%)		
	MVC-COV1901 (N = 341)	Placebo (N = 58)	Total (N = 399)	MVC-COV1901 (N = 341)	Placebo (N = 58)	Total (N = 399)	MVC-COV1901 (N = 340)	Placebo (N = 58)	Total (N = 398)
Grade 1	134 (39.3)	21 (36.2)	155 (38.8)	130 (38.1)	21 (36.2)	151 (37.8)	66 (19.4)	8 (13.8)	74 (18.6)
Grade 2	26 (7.6)	5 (8.6)	31 (7.8)	13 (3.8)	1 (1.7)	14 (3.5)	18 (5.3)	4 (6.9)	22 (5.5)
Grade 3	2 (0.6)	0	2 (0.5)	0	0	0	2 (0.6)	0	2 (0.5)
**Malaise/Fatigue**	105 (30.8)	14 (24.1)	119 (29.8)	78 (22.9)	9 (15.5)	87 (21.8)	63 (18.5)	8 913.8)	71 (17.8)
Grade 1	90 (26.4)	12 (20.7)	102 (25.6)	70 (20.5)	9 (15.5)	79 (19.8)	54 (15.9)	6 (10.3)	60 (15.1)
Grade 2	14 (4.1)	2 (3.4)	16 (4.0)	8 (2.3)	0	8 (2.0)	8 (2.4)	2 (3.4)	10 (2.5)
Grade 3	1 (0.3)	0	1 (0.3)	0	0	0	1 (0.3)	0	1 (0.3)
**Myalgia**	81 (23.8)	13 (22.4)	94 (23.6)	66 (19.4)	10 (17.2)	76 (19.0)	28 (8.2)	4 (6.9)	32 (8.0)
Grade 1	71 (20.8)	11 (19.0)	82 (20.6)	62 (18.2)	9 (15.5)	71 (17.8)	21 (6.2)	3 (5.2)	24 (6.0)
Grade 2	10 (2.9)	2 (3.4)	12 (3.0)	4 (1.2)	1 (1.7)	5 (1.3)	7 (2.1)	1 (1.7)	8 (2.0)
**Headache**	58 (17.0)	13 (22.4)	71 (17.8)	49 (14.4)	9 (15.5)	58 (14.5)	27 (7.9)	6 (10.3)	33 (8.3)
Grade 1	53 (15.5)	10 (17.2)	63 (15.8)	47 (13.8)	9 (15.5)	56 (14.0)	23 (6.8)	3 (5.2)	26 (6.5)
Grade 2	4 (1.2)	3 (5.2)	7 (1.8)	2 (0.6)	0	2 (0.5)	3 (0.9)	3 (5.2)	6 (1.5)
Grade 3	1 (0.3)	0	1 (0.3)	0	0	0	1 (0.3)	0	1 (0.3)
**Diarrhoea**	44 (12.9)	5 (8.6)	49 (12.3)	34 (10.)	4 (6.9)	38 (9.5)	18 (5.3)	2 (3.4)	20 (5.0)
Grade 1	41 (12.0)	5 (8.6)	46 (11.5)	34 (10.)	4 (6.9)	38 (9.5)	15 (4.4)	2 (3.4)	17 (4.3)
Grade 2	3 (0.9)	0	3 (0.8)	0	0	0	3 (0.9)	0	3 (0.8)

	After any dose, n (%)			After first dose, n (%)			After second dose, n (%)		
	MVC-COV1901 (N = 341)	Placebo (N = 58)	Total (N = 399)	MVC-COV1901 (N = 341)	Placebo (N = 58)	Total (N = 399)	MVC-COV1901 (N = 340)	Placebo (N = 58)	Total (N = 398)
**Nausea/Vomiting**	22 (6.5)	5 (8.6)	27 (6.8)	17 (5.0)	2 (3.4)	19 (4.8)	6 (1.8)	3 (5.2)	9 (2.3)
Grade 1	18 (5.3)	4 (6.9)	22 (5.5)	15 (4.4)	2 (3.4)	17 (4.3)	4 (1.2)	2 (3.4)	6 (1.5)
Grade 2	4 (1.2)	1 (1.7)	5 (1.3)	2 (0.6)	0	2 (0.5)	2 (0.6)	1 (1.7)	3 (0.8)
**Fever**	4 (1.2)	0	4 (1.0)	3 (0.9)	0	3 (0.8)	1 (0.3)	0	1 (0.3)
Grade 1	4 (1.2)	0	4 (1.0)	3 (0.9)	0	3 (0.8)	1 (0.3)	0	1 (0.3)

Supplementary Table 4. Summary of unsolicited adverse events and other adverse events

	After any dose, n (%)		
	MVC-COV1901 (N = 341)	Placebo (N = 58)	Total (N = 399)
Unsolicited AEs	84 (24.6)	20 (34.5)	104 (26.1)
Related unsolicited AEs	31 (9.1)	7 (12.1)	38 (9.5)
Unsolicited AEs ≥ Grade 3	6 (1.8)	2 (3.4)	8 (2.0)
Unsolicited AEs ≥ Grade 3 unrelated to study treatment	6 (1.8)	1 (1.7)	8 (2.0)
Related unsolicited AEs ≥ Grade 3	0	1 (1.7)	0
SAEs	0	0	0
Related SAEs	0	0	0
AESI	0	0	0
VAED	0	0	0
AEs leading to study intervention discontinuation	0	0	0
AEs leading to study withdrawal	0	0	0
Death	0	0	0

Abbreviations: AE = adverse event; AESI = adverse events of special interest; N = number of participants in the population; n = number of participants with events; SAE = serious adverse event; VAED = vaccine-associated enhanced disease

Supplementary Table 5. Summary of GMT and GMT ratio of live SARS-COV-2 neutralizing antibody titer by treatment group (IU/mL)

Per protocol set (PPS)<br>Visit 2 (Day 1)	MVC-COV1901 (N=334)	Placebo (N=46)	Ratio MVC/Placebo	P-value
**Median (IQR)**	5.04 (0)	5.04 (0)		
**Q1 ~ Q3**	5.04 ~ 5.04	5.04 ~ 5.04		
**Min ~ Max**	5.04 ~ 5.04	5.04 ~ 5.04		
**GMT**<br>95% CI	5.04<br>5.04 ~ 5.04	5.04<br>5.04 ~ 5.04	1.00<br>1.00 ~ 1.00	N/A
**Per protocol set (PPS)<br>Visit 6 (Day 57)**	**MVC-COV1901 (N=334)**	**Placebo (N=46)**	**Ratio MVC/Placebo**	**P-value**
**Median (IQR)**	643.62 (619.59)	5.04 (0)		
**Q1 ~ Q3**	429.67 ~ 964.11	5.04 ~ 5.04		
**Min ~ Max**	136.20 ~ 3466.39	5.04 ~ 787.75		
**GMT**<br>95% CI	648.47<br>608.62 ~ 690.93	5.63<br>4.51 ~ 7.02	115.18<br>95.4 ~ 139.2.	P < 0.001*

*Two sample t-test

Supplementary Table 6. Summary of GMT and GMT ratio of anti-S-2P IgG titer by treatment group (BAU/mL)

Per protocol set (PPS)<br>Visit 2 (Day 1)	MVC-COV1901 (N=334)	Placebo (N=46)	Ratio MVC/Placebo	P-value
**Median (IQR)**	4.56 (0)	4.56 (0)		
**Q1 ~ Q3**	4.56 ~ 4.56	4.56 ~ 4.56		
**Min ~ Max**	4.56 ~ 36.02	4.56 ~ 22.34		
**GMT**<br>95% CI	4.70<br>4.60 ~ 4.80	4.82<br>4.45 ~ 5.22	0.98<br>0.90 ~ 1.06	0.5557*
**Per protocol set (PPS)<br>Visit 4 (Day 29)**	**MVC-COV1901 (N=334)**	**Placebo (N=46)**	**Ratio MVC/Placebo**	**P-value**
**Median (IQR)**	148.38 (181.76)	4.56 (0)		
**Q1 ~ Q3**	71.59 ~ 253.35	4.56 ~ 4.56		
**Min ~ Max**	10.67 ~ 2810.69	4.56 ~ 24.44		
**GMT**<br>95% CI	136.00<br>122.85 ~ 150.55	4.90<br>4.49 ~ 5.35	27.76<br>24.30 ~ 31.71	P < 0.0001*
**Per protocol set (PPS)<br>Visit 6 (Day 57)**	**MVC-COV1901 (N=334)**	**Placebo (N=46)**	**Ratio MVC/Placebo**	**P-value**
**Median (IQR)**	1637.72 (1548.30)	4.56 (0)		
**Q1 ~ Q3**	1024.54 ~ 2572.84	4.56 ~ 4.56		
**Min ~ Max**	281.26 ~ 8530.76	4.56 ~ 7640.28		
**GMT**<br>95% CI	1631.96<br>1514.93 ~ 1758.04	6.42<br>4.24 ~ 9.71	254.37<br>167.08 ~ 387.25	P < 0.0001*

*Two sample t-test

Supplementary Table 7. Seroconversion rate based on neutralizing antibody titers and anti-S-2P IgG titers by treatment group

Per protocol set (PPS)<br>Visit 4 (Day 29) (IgG)	MVC-COV1901 (N=334)	Placebo (N=46)	Difference<br>MVC/Placebo	P-value
**SCR, n (%)**<br>95% CI	325 (97.3%)<br>94.95 ~ 98.76%	0 (0%)	97.31%<br>95.57 ~ 99.04%	P < 0.0001**
**Per protocol set (PPS)<br>Visit 6 (Day 57) (Neutralization assay)**	**MVC-COV1901 (N=334)**	**Placebo (N=46)**	**Difference<br>MVC/Placebo**	**P-value**
**SCR, n (%)**<br>95% CI	334 (100%)<br>98.90 ~ 100.00%	1 (2.2%)<br>0.06 ~ 11.53%	97.83%<br>93.61 ~ 102.04%	P < 0.0001**
**Per protocol set (PPS)<br>Visit 6 (Day 57) (IgG)**	**MVC-COV1901 (N=334)**	**Placebo (N=46)**	**Difference<br>MVC/Placebo**	**P-value**
**SCR, n (%)**<br>95% CI	334 (100%)<br>98.90 ~ 100.00%	2 (4.3%)<br>0.53 ~ 14.84%	95.65%<br>89.76 ~ 101.55%	P < 0.0001**

* Exact CI of binomial proportion of #

**Chi-square test
