# Supplementary material for: Safety and immunogenicity of SARS-CoV-2 vaccine MVC-COV1901 in Taiwanese adolescents: a randomized phase 2 trial
Source: NPJ Vaccines. 2022 Dec 16;7:165. doi: 10.1038/s41541-022-00589-4 (PMC9755761; doi:10.1038/s41541-022-00589-4)
Supplement: Supplementary file 2 — REPORTING SUMMARY [file 41541_2022_589_MOESM2_ESM.pdf]

nature portfolio

Corresponding author(s): Professor Li-Min Huang

Last updated by author(s): Nov 16, 2022

Reporting Summary

Nature Portfolio wishes to improve the reproducibility of the work that we publish. This form provides structure for consistency and transparency in reporting. For further information on Nature Portfolio policies, see our Editorial Policies and the Editorial Policy Checklist.

Statistics

For all statistical analyses, confirm that the following items are present in the figure legend, table legend, main text, or Methods section.

n/a	Confirmed	
☐	☒	The exact sample size (*n*) for each experimental group/condition, given as a discrete number and unit of measurement
☒	☐	A statement on whether measurements were taken from distinct samples or whether the same sample was measured repeatedly
☐	☒	The statistical test(s) used AND whether they are one- or two-sided<br>*Only common tests should be described solely by name; describe more complex techniques in the Methods section.*
☒	☐	A description of all covariates tested
☐	☒	A description of any assumptions or corrections, such as tests of normality and adjustment for multiple comparisons
☐	☒	A full description of the statistical parameters including central tendency (e.g. means) or other basic estimates (e.g. regression coefficient) AND variation (e.g. standard deviation) or associated estimates of uncertainty (e.g. confidence intervals)
☒	☐	For null hypothesis testing, the test statistic (e.g. *F*, *t*, *r*) with confidence intervals, effect sizes, degrees of freedom and *P* value noted<br>*Give P values as exact values whenever suitable.*
☒	☐	For Bayesian analysis, information on the choice of priors and Markov chain Monte Carlo settings
☒	☐	For hierarchical and complex designs, identification of the appropriate level for tests and full reporting of outcomes
☒	☐	Estimates of effect sizes (e.g. Cohen's *d*, Pearson's *r*), indicating how they were calculated

Our web collection on statistics for biologists contains articles on many of the points above.

Software and code

Policy information about availability of computer code

Data collection	N/A
Data analysis	N/A

For manuscripts utilizing custom algorithms or software that are central to the research but not yet described in published literature, software must be made available to editors and reviewers. We strongly encourage code deposition in a community repository (e.g. GitHub). See the Nature Portfolio guidelines for submitting code & software for further information.

Data

Policy information about availability of data

All manuscripts must include a data availability statement. This statement should provide the following information, where applicable:

- Accession codes, unique identifiers, or web links for publicly available datasets
- A description of any restrictions on data availability
- For clinical datasets or third party data, please ensure that the statement adheres to our policy

Data and clinical trial protocol are available from the authors upon reasonable request.

Human research participants

Policy information about studies involving human research participants and Sex and Gender in Research.

Reporting on sex and gender	Sex and gender were not differentiated during the study. The "Gender" defined in the study was based on the government-issued personal identification card of the individual participants.
Population characteristics	The adolescents ranged from 12 to less than 18 years of age, and the young adults ranged from 20 to 30 years of age. The participants for the final analysis were negative for COVID-19 throughout the study period and have not had any prior COVID-19 infections. The participants were also naive for any COVID-19 vaccination prior to the study.
Recruitment	Recruitment was driven by recruitment poster placed in hospitals and word-of-mouth. The recruitment is an open to all interested applicants and no pre-screening was employed to minimize selection bias.
Ethics oversight	Taiwan Food and Drug Administration (TFDA)

Note that full information on the approval of the study protocol must also be provided in the manuscript.

Field-specific reporting

Please select the one below that is the best fit for your research. If you are not sure, read the appropriate sections before making your selection.

☒ Life sciences ☐ Behavioural & social sciences ☐ Ecological, evolutionary & environmental sciences

For a reference copy of the document with all sections, see nature.com/documents/nr-reporting-summary-flat.pdf

Life sciences study design

All studies must disclose on these points even when the disclosure is negative.

Sample size	The sample size for this study is not based on a formal statistical power calculation. The sample size is considered sufficient to evaluate the objectives of the study. With 330 participants receiving MVC-COV1901, there are 96.4%, 80.9% and 28.1% probabilities to observe at least 1 subject with an AE if the true incidence of the AE are 1.0%, 0.5% and a 0.1%, respectively.
Data exclusions	A total of 19 participants were excluded from the final per protocol set analysis. The reasons for exclusion include violation of the exclusion criteria, positive for COVID-19 infection, and/or use of prohibited medication during the study period.
Replication	N/A
Randomization	All participants were centrally assigned to randomized study intervention using an interactive web response system (IWRS). Before the study was initiated, the log-in information and directions for the IWRS were provided to each site.
Blinding	This was a double-blind study in which participants and investigators were blinded to study intervention. The IWRS was programmed with blind-breaking instructions and in case of an emergency, the investigator had the sole responsibility for determining if unblinding of participants' intervention assignment was warranted. For the interim analysis, an independent, unblinded team of the CRO consisting of personnel representing relevant functions, including statistics, programming, and report writing, was involved in the activity of the interim analysis. All activities of the unblinded team were separated from the main blinded team of the study. The Sponsor was blinded until the time of the interim analysis.

Reporting for specific materials, systems and methods

We require information from authors about some types of materials, experimental systems and methods used in many studies. Here, indicate whether each material, system or method listed is relevant to your study. If you are not sure if a list item applies to your research, read the appropriate section before selecting a response.

Materials & experimental systems

n/a	Involved in the study
☒	☐ Antibodies
☒	☐ Eukaryotic cell lines
☒	☐ Palaeontology and archaeology
☒	☐ Animals and other organisms
☐	☒ Clinical data
☒	☐ Dual use research of concern

Methods

n/a	Involved in the study
☒	☐ ChIP-seq
☒	☐ Flow cytometry
☒	☐ MRI-based neuroimaging

Clinical data

Policy information about clinical studies

All manuscripts should comply with the ICMJE guidelines for publication of clinical research and a completed CONSORT checklist must be included with all submissions.

Clinical trial registration: NCT04951388

Study protocol: Can be provided upon reasonable request

Data collection: Data were collected on-site at the five hospital/medical center in Taiwan as described in the manuscript. The study began to screen participants since July 21, 2021 and the manuscript is based on the study data collected up to December 22, 2021.

Outcomes: The primary safety outcome included the occurrence rate of solicited (local and systemic) AEs, unsolicited AEs, AESI, VAED, and SAE from Visit 2 (Day 1) to Visit 6 (28 days after second dose of study intervention). The primary immunogenicity outcomes were neutralizing antibody titers and seroconversion rate (SCR) against live SARS-CoV-2 virus of MVC-COV1901 in adolescents as compared to young adult vaccinees at Visit 6 (Day 57).
The secondary safety outcome included the occurrence rate of ≥ Grade 3 AE, AESI, VAED, and SAE over the whole study period i.e. from Day 1 to 180 days after the second vaccination (Day 209). For secondary immunogenicity outcomes, comparisons of MVC-COV1901 against placebo were performed by measuring and expressing antigen-specific immunoglobulin titers and neutralizing antibody titers in samples taken at Visit 4 (28 days after the first dose of study intervention), Visit 6 (28 days after the second dose of study intervention), Visit 8 (90 days after the second dose of study intervention) and Visit 9 (209 days after the second dose of study intervention).
